# Three Reasons for Playing the Tennis Forehand in Square Stance

**DOI:** 10.3390/jfmk10020215

**Published:** 2025-06-05

**Authors:** Lucio Caprioli, Francesca Campoli, Cristian Romagnoli, Ida Cariati, Saeid Edriss, Elvira Padua, Vincenzo Bonaiuto, Giuseppe Annino

**Affiliations:** 1 Sports Engineering Laboratory, Department of Industrial Engineering, University of Rome Tor Vergata, 00133 Rome, Italy; lucio.caprioli@uniroma2.it (L.C.); saeid.edriss@alumni.uniroma2.eu (S.E.); vincenzo.bonaiuto@uniroma2.it (V.B.); 2 Department of Human Science and Promotion of Quality of Life, San Raffaele Rome University, 00166 Rome, Italy; cristian.romagnoli@uniroma5.it (C.R.); elvira.padua@uniroma5.it (E.P.); 3 Department of Systems Medicine, “Tor Vergata” University of Rome, Via Montpellier 1, 00133 Rome, Italy; 4 Human Performance Laboratory, Centre of Space Bio-Medicine, Department of Medicine Systems, University of Rome Tor Vergata, 00133 Rome, Italy; giuseppe.annino@uniroma2.it

**Keywords:** tennis, neutral stance, open stance, performance, injury prevention, hip impingement

## Abstract

This study aims to compare the effectiveness of tennis forehand shots played by competitive athletes in open and square stances in terms of performance: (1) ball speed, (2) accuracy, and (3) gesture economy. This is with the aim of preventing the excessive wear and tear of the athlete’s musculoskeletal structures. Methods: Between October 2024 and January 2025, forty-two healthy players were involved in the study. Eighty forehands were played by each subject with open and square stances in lateral and diagonal-inside running structured situations. The ball’s speed, shot accuracy, and the athlete’s heart rate were acquired. Kruskal–Wallis’s and Dunn post hoc tests were used to compare the effect of stance, tactical situation, gender, and player’s flexibility on these performance variables. The Wilcoxon signed-rank *t*-test was applied to compare each of the two types of stances. Results: Square stance consistently resulted in significantly higher ball speeds in both lateral running (Δ_Median_: 6 km/h) and diagonal-inside running (Δ_Median_: 4 km/h), while the differences in accuracy and metabolic demand were not significant overall. Conclusions: This study found that the square stance technique provides a clear advantage in terms of ball speed. Although the higher accuracy found was not significant, the small difference in metabolic effort was. Overall, the benefits reported seem to make the square stance the preferable choice.

## 1. Introduction

In tennis, the transfer of the kinetic chain starts from the legs [1,2]. A proper stance, i.e., the position of the feet in relation to the ball, is a key element in technique. In particular, in rebound shots, a good balance during the execution of the gesture can allow the transfer of more energy to the ball [1,3,4]. The stances are mainly classified into square (or neutral), semi-open, and open stances [5,6]. The square stance is played with feet aligned perpendicular to the net, and in the open, the feet are aligned parallel to each other (the player is fully facing the net). The intermediate positions between these two stances are called semi-open stances [7,8,9]. They are adapted within various tactical contexts, such as offensive or defensive stances and dynamic stance types [10]. The non-dominant foot generally has a fulcrum function in the forehand, while the dominant one is the limb mainly involved in pushing.

The open stance forehand has become a much-played stroke in more recent times, considering the long history of this sport [11,12]. It became popular with several top players, such as Andre Agassi and Gabriela Sabatini, in the 1990s. The new century has seen a growing interest in and use of this stance, as opposed to the more traditional square stance [13,14,15]. This change in trend has sparked debate among experts, leading to the need for an in-depth analysis of the advantages and disadvantages of each technique in modern tennis. Several factors may have contributed to this evolution of the game. Indeed, hypotheses favoring the open stance were supported by a study [7] that argued that this stance offers more power in strokes, allowing athletes to generate more spin and reach wider angles. Another aspect to consider is that since the player faces the opponent in an open stance, it may favor the recovery of the position, which may be the more comfortable choice. However, the results in the literature are sometimes discordant [7,9], and it is unclear what the real advantages of using one technique over the other are.

On the other hand, it has been theorized that using the open stance may increase the risk of spondylolisthesis [16] in a discipline that is already very traumatic at the spine level [17,18,19]. In the shots played with open stance by 14 collegiate tennis players, the mean normalized surface electromyography (EMGs) of the erector spinae was significantly larger than the rectus abdominis and external oblique, which is congruent with observations of strength imbalances and an increasing incidence of low back injuries in tennis [12]. No evidence at the upper limb level supported the hypothesis that the open stance technique creates a greater load than the square stance technique [13]. On the contrary, plantar pressure values were evaluated during forehand strokes in open and square stances (topspin) in elite female tennis players, finding that maximal and mean forces were significantly larger during open compared to square stance for the dominant foot [20]. Other studies [8,21] have suggested that using the open stance, particularly the defensive open stance, potentially increases the risk of hip overuse injuries, such as posterior–superior impingement, and knee injuries. Indeed, increases in the vertical ground reaction force [22], maximum knee flexion and abduction angles, knee flexion–extension range, peak knee compressive, distraction, medial forces, peak knee abduction, external rotation torques, and the combination of extreme hip flexion, abduction, and external rotation movements were found in this technique [6,8,21]. Therefore, they encourage the use of the neutral stance [16,21].

Understanding the differences between the two stances and their effects on technique and performance is crucial for coaches and players who wish to optimize their game. Although, from what emerges in the literature, the traditional square stance has to be preferred for a conservative approach to injury prevention, and this is certainly true in amateur tennis, there is still little clarity regarding the effectiveness of the two types of stances. This study aims to compare the efficacy of the two techniques in terms of performance: (1) ball speed, (2) accuracy, and (3) gesture economy. The choice of these three indicators stems from the demands of the performance model of this sport. Indeed, every tennis player wants to play strongly with precision and minimal effort [10].

The first relevant question we aim to answer is whether the square stance in both offensive (inside the court) situations and at the baseline (lateral running) allows for greater ball speed. The second relevant question asks whether the square stance provides higher stroke accuracy. The third question asks whether the forehand shot played in the square stance engages the athlete more metabolically than in the open stance.

## 2. Materials and Methods

All the measurements were conducted between October 2024 and January 2025 in several tennis clubs in Italy, in indoor and outdoor courts on windless days (less than 3 km/h and without variation during the performances of the same player, according to data acquired from the meteorological stations). The playing surfaces on which the tests were conducted were 60% hard, 21% clay, and 19% grass, with a similar proportion to the distribution of playing surfaces in tennis tournaments [23,24].

### 2.1. Data Collection

The study involved forty-two tennis players (*n* = 42: 17 females, 25 males; stature: 174 ± 9 cm; body mass: 65 ± 10 kg) from 13 to 58 years old. All subjects had practiced the discipline continuously in the last five years, were ranked, and played tournaments competitively at national and international levels. Most of the subjects were of Italian nationality, with four participants from Greece, Turkey, Morocco, and the United States. They used their own equipment for the experiments and wore a Polar H10 chest belt (Polar Electro, Kempele, Finland). The flexibility of the players’ posterior chain muscles was assessed through the Sit and Reach test [25] using a portable version of the instrument [26]. A division into two flexibility level classes was made, where all the results greater than zero were considered good flexibility, and those lower were considered low. Odea Win pressurized balls, approved by the International Tennis Federation [27], were opened and used for tests on the same day of the experiment.

All recruited subjects declared that they were in good health, had no recent injuries and gave written informed consent to data processing for research purposes. The research was approved by the Internal Research Board of “Tor Vergata” University of Rome. All procedures were carried out in accordance with the Declaration of Helsinki.

### 2.2. Performance Assessment

After a proper twenty-minute physical and technical warm-up, the subjects performed two sets of ten forehand shots for the two stance types and two different tactical situations, namely lateral and diagonal-inside running, participating crosswise in four groups (conditions): lateral square stance (LSS), lateral open stance (LOS), diagonal-inside square stance (DSS) and diagonal-inside open stance (DOS). This was structured as shown in Table 1.

**Figure 1:**
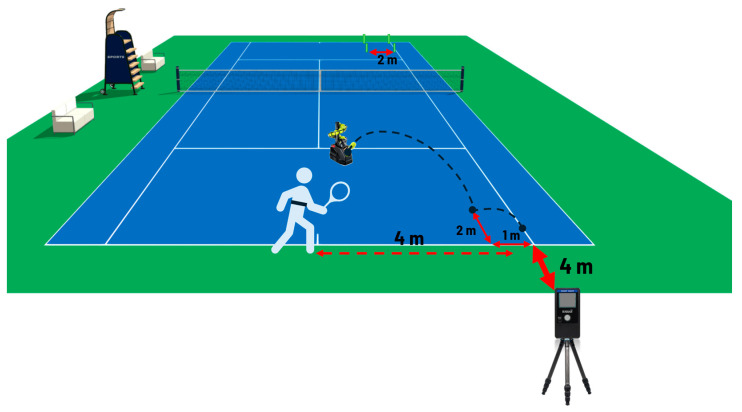
Positioning of the equipment in the lateral running situation. The speed radar was placed on a tripod 4 m behind the intersection of the baseline and the right sideline and at a 1.10 m height from the ground; a square-shaped target with 2 m sides delimited with four cans on the opposite side of the court; the ball-throwing machine at 1.5 m from the intersection of the service lines, so that the ball bounces 2 m inside the court and 1 m from the sideline.

They each received 80 balls from a ball-throwing machine that were easy to handle (TEKNIGOO—China). No technical feedback was provided to participants during the tests, and the instruction they received was to play all the shots fast and precisely in the target area (Figure 1). The interval between each ball was six seconds, and the rest between the sets was ninety seconds. In both tactical situations, a square-shaped target with 2 m sides was on the opposite side of the court at the intersection of the baseline and the sideline, as shown in Figure 1. In the lateral running situation, the ball-throwing machine was positioned at 1.5 m from the intersection of the service lines so that the ball bounced 2 m inside the court and 1 m from the sideline (Figure 1). The player started with both feet behind the baseline, to the left of the mid-point (the center mark), and had to return after each shot to the starting position, covering about 4 m with each change in direction [28]. A speed radar model, namely the Smart Coach Radar, with an operating frequency of 24.125 GHz (Pocket Radar Inc., Santa Rosa, CA, USA) was placed on a tripod placed 4 m behind the intersection of the baseline and the right sideline and at a 1.10 m height from the ground (Figure 1) [29].

**Figure 2:**
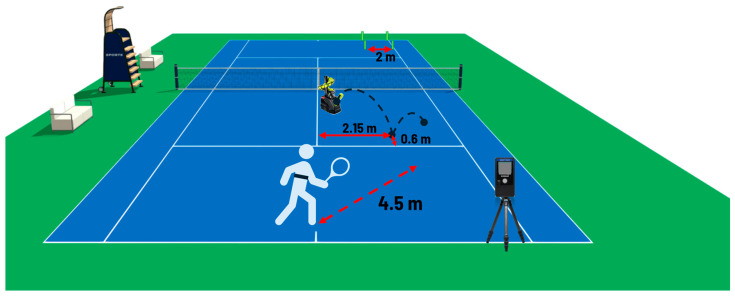
Positioning of the equipment in the diagonal-inside running situation. The speed radar was placed on a tripod on the baseline close to the right sideline and at a 1.10 m height from the ground; a square-shaped target with 2 m sides delimited with four cans on the opposite side of the court; the ball-throwing machine at 2.5 m inside the serve box, so that the ball bounces 0.6 m inside the box and 2.15 m from the service central line.

**Figure 3:**
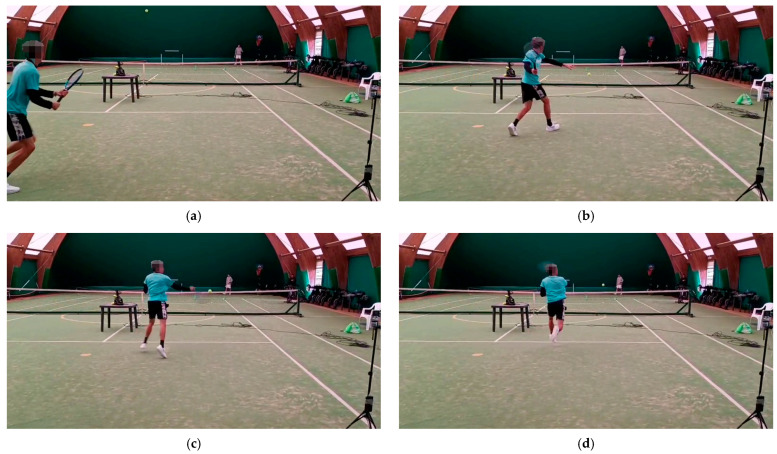
The sequence of an open-stance forehand played in a diagonal-inside running situation by a participant: (**a**) starting point; (**b**) approaching the ball; (**c**) impact; (**d**) final.

In the diagonal-inside running series, the ball-throwing machine was placed at 2.5 m inside the serve box area, so the ball bounced 0.6 m inside the box and 2.15 m from the central service line (Figure 2). The player started 1 m inside the court and had to return after each shot to the starting position, covering about 4.5 m on each change in direction [28]. The speed radar was placed on a tripod on the baseline, near the right sideline, and at a 1.10 m height from the ground. An evaluator checked the accuracy of the shot. The Polar H10 chest belt acquired heart rate, heart rate variability [30,31,32], and accelerometer data [33] set to a sampling frequency of 100 Hz. Figure 3 shows some frames of the execution of an open-stance forehand played in a diagonal-inside running situation by a participant of the study.

### 2.3. Exclusion Criteria

The heart rate data were compromised in some subjects’ repetitions due to the movement of the heart rate band, which was probably attributable to a maladjustment. To prevent these data from compromising the goodness-of-effect of the survey, the heart rate data from these series were not considered in the study. Therefore, the heart rate data of thirty-nine players were analyzed for LOS and LSS situations and those of thirty-five were analyzed for DOS and DSS. Although this study was conducted on experienced tennis players at the national and international level, a qualified technician verified the correct position, and a posteriori exclusion of executions deemed incorrect or affected by the player’s personalism was carried out. Indeed, the performance of the diagonal-inside running exercise in two subjects (#22 and #41) was found to be poorly performed; we therefore excluded these trials from the analysis.

### 2.4. Statistical Analysis

The Shapiro–Wilk test was used to validate the assumption of normality. As not all data followed a normal distribution and to increase the power of the statistics, nonparametric tests were used for inferences. The Intraclass Correlation Coefficient (ICC) [34,35,36] and Spearman’s correlation (ρ) [37,38] were calculated to determine the reliability between the two sets of measures for the four tactical situations. The effect sizes (ESs) given by the Rank-Biserial Correlation were calculated between the first and second set averages [39], where a small ES was 0.10–0.29, moderate was 0.30–0.49, and large was >0.50 [40,41]. The sample size and the statistical power were calculated through the Jasp power analysis module based upon “jpower” by Richard Moorey [42]. It needed a sample size of 27 in each group to reliably (with a probability greater than or equal to 0.9) detect an effect size of |δ| ≥ 0.9, assuming a two-sided criterion for detection that allows for a maximum Type I error rate of α = 0.05. The Passing–Bablok regression was used to assess systematic differences between the two set trials, calculating the slope (B) and intercept (A) along with their 95% confidence intervals of a regression equation [43]. Since a deviation from the multivariate normality was found by the Shapiro–Wilk test, Kruskal–Wallis and Dunn post hoc tests were used for comparisons of the dependent variables (ball speed, accuracy, and heart rate) between the two types of stances, tactical situation, gender and flexibility level of the players [44,45,46]. In addition, the Wilcoxon signed-rank *t*-test was used to compare each performance variable (ball speed, accuracy, and heart rate) between the two types of stances. A statistical analysis of the data was carried out with Jasp software (Version 0.18.3) [47] and R software (Version 4.4.2) [48].

## 3. Results

### 3.1. Reliability

The test–retest values of the median, inter-quartile range (IQR), ICC, 95% confidence interval (CI) for ICC, Spearman’s correlation (ρ), and the ES relative to the ball speed, accuracy ratio, and maximum heart rate in the four tactical situations performed in the two sets of measurements are reported in Table 2.

Table 3 shows the Passing–Bablok regression of the ball speed, accuracy ratio, and maximum heart rate in the four tactical situations: LOS, lateral open stance; LSS, lateral square stance; DOS, diagonal-in open stance; DSS, diagonal-in square stance.

### 3.2. Performance Assessment

Ball speed and accuracy data from a total of 3280 shots played by the forty-two subjects in the four different tactical situations (LOS, Lateral Open Stance; LSS, Lateral Square Stance; DOS, Diagonal-in Open Stance; DSS, Diagonal-in Square Stance) were analyzed. Within all measurements, the highest recorded ball speed was 167 km/h in a forehand executed by a male player in the DSS. The shot played inside the target at the highest speed was a 164 km/h right executed in the LSS. Among female subjects, the fastest shot directed into the area reached 151 km/h in the DSS. These maximum values were recorded in both males and females in indoor courts. In contrast, regarding outdoor play, the highest speeds recorded were 142 km/h among women in DSS and 153 km/h among men in LSS. The best sets of shots in terms of accuracy were performed by a girl (9/10) and the boys (8/10) in the DOS.

The forty-two subjects’ ball speed and accuracy ratio in the four tactical situations are reported in the Supplementary Materials [49]. The Kruskal–Wallis test showed that stance, flexibility, and gender factors significantly affected the ball speed, as confirmed by the Dunn post hoc results reported in Table 4.

**Figure 4:**
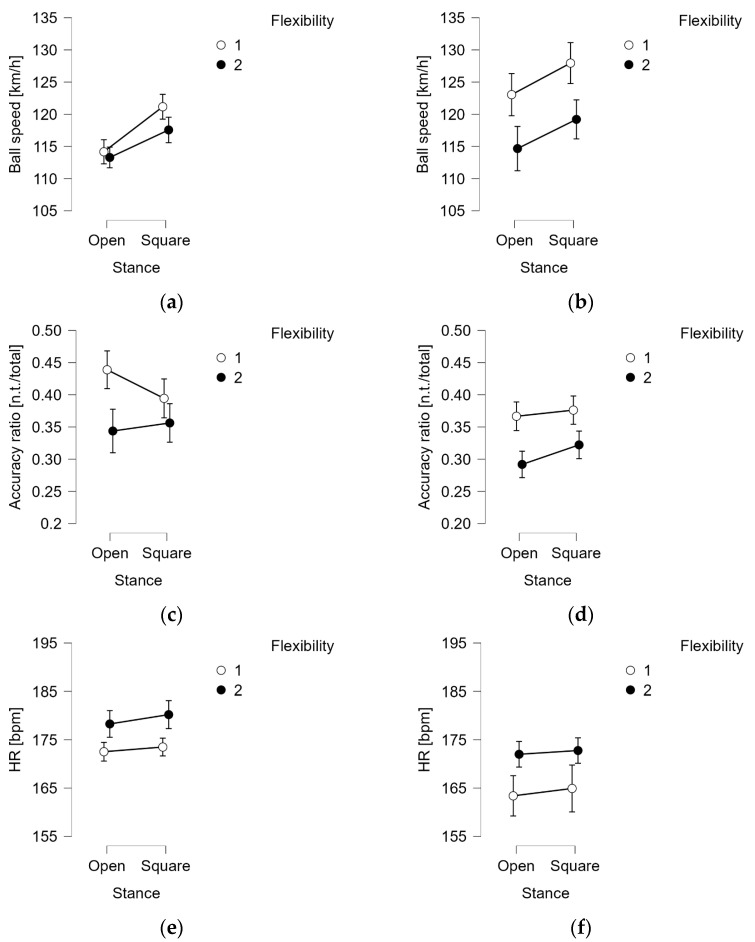
Descriptive plots of the comparisons of each of those dependent variables: ball speed in females (**a**) and in males (**b**); accuracy in females (**c**) and in males (**d**); and heart rate in females (**e**) and in males (**f**) between the two types of stances, tactical situation and flexibility level of the players.

Figure 4 shows some descriptive plots of the comparisons of ball speed in females (Figure 4a) and males (Figure 4b) between the two types of stances and the flexibility level of the players. Comparisons of the accuracy ratio between the tactical situation and flexibility level are shown in Figure 4c (females) and Figure 4d (males). Finally, heart rate comparisons are depicted in Figure 4e,f for females and males, respectively.

It can also be seen that while the type of stance is the most significant factor for ball speed, it does not substantially affect the accuracy and heart rate. At the same time, the tactical situation and gender and flexibility factors are always significant for accuracy (Table 4). Specifically, men played at an average speed of 121 ± 16 km/h and women played at an average speed of 117 ± 8 km/h, while women had a higher average accuracy ratio (Females: 0.39 ± 0.1—Males: 0.34 ± 0.1). Subjects with good flexibility were found to have a higher ball speed (Δ: 6 km/h), accuracy (Δ: 7%), and lower maximum heart rate (Δ: 7 bpm) than those with less flexibility. Table 5 reports the median, IQR, minimum, and maximum values of the ball speed (km/h) and accuracy ratios (number of valid targets/total) achieved when performing the exercises in the four tactical situations. The Wilcoxon *p*-value, effect size, and standard error of the effect size for the comparations between LSS and LOS, as well as the DSS and DOS in ball speed and accuracy ratio, are described in Table 5.

In the lateral running situation, LSS showed a median value that was 6 km/h higher than LOS, which is highly statistically significant according to the Wilcoxon signed-rank *t*-test (*p* < 0.001). A smaller difference, always highly statistically significant, was in the diagonal-inside running forehand, where the median value of the DSS was found to be 4 km/h higher than DOS. The statistical power according to the effect size was higher than 98% in both situations for ball speed.

**Figure 5:**
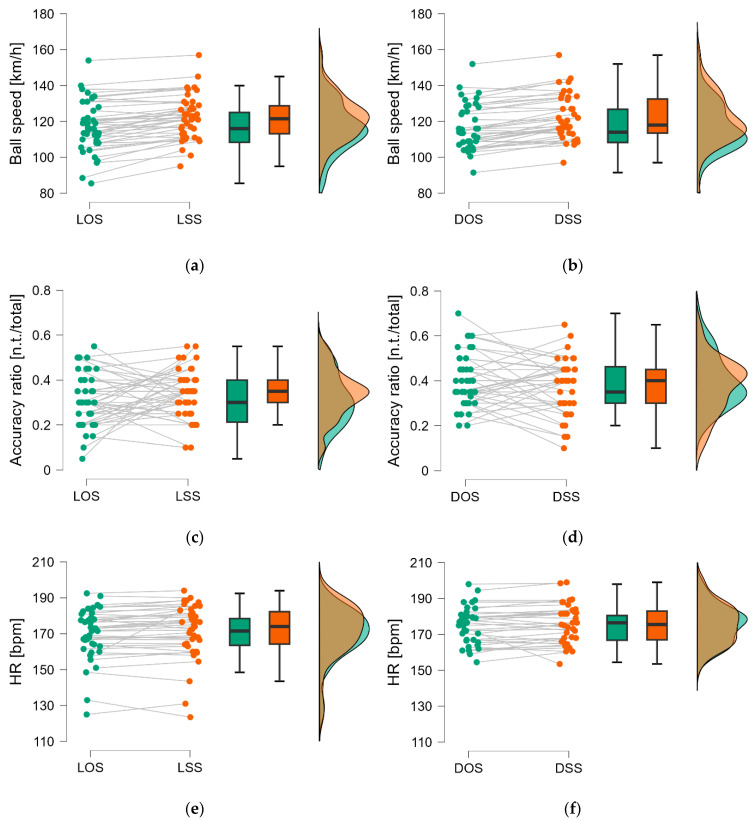
Raincloud plots relative to the comparations of (**a**) lateral open stance (LOS) and lateral square stance (LSS) ball speed; (**b**) diagonal-inside open stance (DOS) and diagonal-inside square stance (DSS) ball speed; (**c**) lateral open stance (LOS) and lateral square stance (LSS) accuracy ratio; (**d**) diagonal-in open stance (DOS) and diagonal-in square stance (DSS) accuracy ratio; (**e**) lateral open stance (LOS) and lateral square stance (LSS) heart rate; (**f**) diagonal-in open stance (DOS) and diagonal-inside square stance (DSS) heart rate. Open stances are represented in green, while square in orange.

In terms of accuracy, the square stance median values were higher than the open stance. However, the difference was not statistically significant (*p* > 0.05), and the statistical power according to the effect size was lower than 15% in both situations. Overall, it is possible to see in the raincloud plots, relative to the comparations of the two different analyzed stance techniques (Figure 5), that the square stance is associated with better performances except in the diagonal-inside accuracy ratio (Figure 5d).

The maximum heart rate (bpm) of the subjects achieved when performing the exercises in the four tactical situations expressed, as the median value of executions, is reported in the Supplementary Materials [49].

Table 5 shows the median values of the maximum heart rate, relative to the whole group (bpm), that were achieved when performing the exercises in the four situations. The Wilcoxon *p*-value, effect size, and standard error of the effect size for the comparations between LSS and LOS, as well as DSS and DOS for the maximum heart rate, are described in Table 5. In the lateral running situation, LSS showed a median value that was two beats per minute higher than LOS (*p* < 0.05). The same difference, but not statistically significant, was in the diagonal-inside running forehand, where the median value of the DSS was found to be higher than DOS. The statistical power according to the effect size was 56% in lateral running and 37% in diagonal inside situations.

The following plots (Figure 5e,f) compare the maximum heart rate reached in the two analyzed stance techniques.

## 4. Discussion

In this study conducted on experienced participants who had been competitively active, both nationally and internationally, in the 5 years before the measurements, the effectiveness of the two stance techniques, namely open and square stance, in two specific game situations was assessed through two standardized exercises: lateral running and diagonal-distance running.

It must be premised that the choice of these two situations occurred in line with the purpose of this investigation, which was to provide clear indications as to the type of support that is preferable when the tennis player has a way and time to choose. In fact, for this reason, the back shift off the court used in defensive situations where the player must adapt as well as he can in a short time [50] was not considered. Furthermore, considering the information that has emerged in the literature, open stance is associated with a greater risk of hip, knee, and ankle injuries, as well as lumbar injuries [12,16]; this is due to greater overload at the dominant lower limb [8,20,21] and more extreme joint angles [6,8,22]. We want to answer the question of what type of stance is appropriate to use to enhance performance in the proposed situations.

The set-to-set repeatability comparison showed that good congruence between the ball speed and maximum heart rate was reached in the two sets, validating the significance of the investigation. However, low levels of reliability were found in the accuracy ratio (Table 2). The Passing and Bablok regressions showed no proportional differences. Indeed, slope B’s CI included 1 [43]; this is except for the heart rate in LSS and accuracy ratio in DOS, where a clear difference was found. No systematic bias was detected (intercept A’s CI includes 0, except for heart rate in LSS) [43], nor were significant deviations from linearity (Table 3). The systematic bias shown by the Passing and Bablok regressions between the two sets of LSS measurements could be caused by the metabolic commitment of the exercise; during the second set of measurements, the subjects were more fatigued, and although statistically significant, the difference in median value was moderate (Δ_Median_: 1 bpm).

The low reliability found in the variable accuracy limits the strength of conclusions in this aspect. This variability in shot accuracy may also be affected by psychological factors such as attention, concentration, self-talk [51], motivation, arousal, self-confidence, and stress management [52,53,54]. Therefore, these results were in line with the authors’ expectations.

To assess the overall effect of the athletes’ stance, tactical situation, gender, and level of flexibility on the numerical performance variables (speed, accuracy, and heart rate), since a deviation from multivariate normality was found, we used Kruskal–Wallis and Dunn post hoc tests to carry out similar inferences and evaluate the effect of each in detail. These showed that stance significantly affects ball speed and not the other two (accuracy and metabolic effort). As expected, the gender factor was significant in all three variables, showing a difference between genders, where males showed a higher ball speed while females showed more accuracy. As for heart rate, although it was found to be different in the two sexes, the result was not considered as there was an average age difference of 3 years between men and women, and this mismatch may have caused bias.

The significant effect of flexibility in each of the three variables could result from the fact that, although having a similar experience and technical level of play, the more flexible subjects might be those with better physical conditioning. This finding may lead one to consider the utility of the Sit and Reach test in tennis and potentially propose flexibility as an indicator of the level of physical training in tennis players, showing it to be correlated with performance in tennis as well [55,56,57]. However, this interpretation is only a suggestion, and the sit-and-reach test alone cannot be considered an indicator of the subject’s overall conditioning level.

The results of this study showed that in both situations, the square stance allows for the generation of a greater ball speed, as shown in Figure 3a,b, with a highly statistically significant difference (Table 4) of 6 km/h in the lateral run and 4 km/h in the diagonal inside move. Regarding accuracy, the square stance median values were higher than the open stance. However, the difference found was not statistically significant. Although the first supported hypothesis regarding ball speed was fully confirmed, the second regarding accuracy cannot be. From the heart rate measurement, it was found that LSS had a slightly higher metabolic commitment (Δ = 2 bpm, *p* < 0.05), as did DSS, although with a statistically insignificant difference. This difference, particularly in the side-stance situation, is probably attributable to the easier recovery of the position when using the open stance compared to the square stance. In fact, in the LOS, the player, during the execution of the shot, is already in a frontal position to the playing court, while in the LSS, an additional step is usually required. However, given the small difference found and the potential measurement bias, this could be contextualized as potentially negligible.

Following these considerations, the performance advantage of the square stance over open, in terms of speed and accuracy, is evident in the face of a greater metabolic commitment in the lateral displacement, which was not found to be considerable. Therefore, the square stance should be chosen whenever the opportunity is presented for performance and injury prevention advantages.

In the last instance, considering that the use of open stances in the game cannot be precluded despite the commitment of the athlete to preferring closed stances, just as much commitment should not be lacking in the methodical and consistent execution of a training program aimed at maintaining a balance in the muscle groups and ensuring daily stretching, which could help reduce stress on the joints and facilitate the resumption of activity in the absence of pain following femoroacetabular impingement [58]. The significant positive effect of flexibility reinforces this good practice in training, which was clear in the players examined.

### Limitations

Although this study was carried out on an adequate sample of experienced subjects from different training centers created by various coaches, which can be considered representative, it still reflects a small sample of the investigated population, and personal differences could occur in players with unexamined playing techniques and personalisms. The nonsignificant difference in accuracy can be considered an additional practical limitation of the study, as it appears that this variable is affected more by several factors than by the stance technique. Due to a concern for the standardization of procedures and the practicality of configuring the radar instrumentation so that it is positioned in the same direction as the shot, only the forehand in the long line direction was examined. Moreover, it must be highlighted that, to ensure standardization, the study was focused on a structured drill that differs from what the player faces in the game. In future developments, additional game situations with different targets may be proposed to investigate possible differences in angle variation and trajectories.

## 5. Conclusions

Despite the practical limitations of this study, such as the low reliability and the modest sample of players who conducted the tests on the various playing surfaces, the collected results have shown, with good statistical power, that the open stance does not offer clear advantages to the tennis player. This study provides evidence to support the use of the square stance technique in tennis, particularly regarding the achievement of a greater ball speed. Although a slight increase in metabolic demand was observed in the forehand played in lateral running, square-stance demonstrates (1) advantages in generating a high ball speed, (2) good accuracy, and (3) an almost negligible difference in metabolic effort. What was found, combined with the potential for a reduced risk of injury, makes the square stance a good choice for players and coaches in all game contexts where the player has time to adjust into this position.

## Figures and Tables

**Table 1:** Forehand assessment protocol structured in four tactical situations: DSS, diagonal-inside square stance; DOS, diagonal-inside open stance; LSS, lateral square stance; LOS, and lateral open stance.

Tactical Situation	Type of Stance	1° Set	2° Set
Lateral	Square	10 shots	10 shots
	Open	10 shots	10 shots
Diagonal in	Square	10 shots	10 shots
	Open	10 shots	10 shots

**Table 2:** Set-to-set repeatability of ball speed, accuracy ratio, and maximum heart rate in the four tactical situations: LOS; LSS; DOS and DSS.

Ball Speed [km/h]									
	Set 1		Set 2						
Measure	Median	IQR	Median	IQR	ICC_3,k_	95% CI	ρ	ES	SE
LOS	115	18	117	21	0.974	0.951 to 0.986	0.928 ***	−0.517	0.182
LSS	120	18	122	17	0.978	0.958 to 0.988	0.957 ***	−0.454	0.182
DOS	111	20	111	20	0.982	0.966 to 0.988	0.932 ***	−0.239	0.206
DSS	116	19	119	17	0.970	0.945 to 0.984	0.904 ***	−0.127	0.200
**Accuracy ratio [n. of valid target/totals]**									
	Set 1		Set 2						
Measure	Median	IQR	Median	IQR	ICC_3,1_	95% CI	ρ	ES	SE
LOS	0.30	0.13	0.30	0.20	0.113	−0.195 to 0.400	0.084	−0.432	0.200
LSS	0.30	0.10	0.30	0.10	0.179	−0.128 to 0.456	0.147	0.220	0.200
DOS	0.40	0.33	0.40	0.20	0.000	−0.301 to 0.301	−0.228	−0.232	0.189
DSS	0.40	0.20	0.30	0.30	0.258	−0.046 to 0.519	0.284	0.292	0.186
**Max Heart Rate [bpm]**									
	Set 1		Set 2						
Measure	Median	IQR	Median	IQR	ICC_3,k_	95% CI	ρ	ES	SE
LOS	170	14	173	18	0.936	0.883 to 0.965	0.917 ***	−0.758	0.197
LSS	174	22	175	18	0.955	0.917 to 0.975	0.962 ***	−0.652	0.191
DOS	174	15	176	17	0.818	0.687 to 0.898	0.778 ***	−0.553	0.221
DSS	174	18	177	20	0.828	0.701 to 0.903	0.829 ***	−0.488	0.206

*** *p*-value < 0.001. LOS, lateral open stance; LSS, lateral square stance; DOS, diagonal-in open stance; DSS, diagonal-in square stance. IQR, inter-quartile range; ICC_3,k_, intraclass correlation coefficient; 95% CI, 95% confidence interval (CI) for ICC; ρ Spearman’s correlation coefficient; ES, effect size by the matched rank biserial correlation; SE, standard error of the effect size.

**Table 3:** Passing and Bablok regression of the ball speed, accuracy ratio, and maximum heart rate in the four tactical situations: LOS; LSS; DOS and DSS.

Ball Speed [km/h]					
	Systematic Differences		Proportional Differences		Linear Model Validity
Measure	Intercept A	95% CI	Slope B	95% CI	Cusum Test for Linearity
LOS	−2.260	−14.951 to 10.102	1.002	0.892 to 1.119	*p* = 0.97
LSS	5.577	−9.309 to 13.953	0.942	0.871 to 1.067	*p* = 0.80
DOS	−5.625	−24.783 to 3.836	1.043	0.959 to 1.204	*p* = 0.19
DSS	1.746	−14.053 to 22.689	0.988	0.8000 to 1.1108	*p* = 0.67
**Accuracy ratio [n. of valid target/totals]**					
	Systematic differences		Proportional differences		Linear model validity
Measure	Intercept A	95% CI	Slope B	95% CI	Cusum test for linearity
LOS	−0.100	−0.500 to 0.100	1.000	0.500 to 2.167	*p* = 0.39
LSS	0.000	−0.400 to 0.200	1.000	0.500 to 2.375	*p* = 0.71
DOS	−0.450	−1.650 to 0.050	2.000	-	*p* = 0.38
DSS	0.075	−0.300 to 0.233	0.750	0.500 to 2.000	*p* = 0.05
**Max Heart Rate [bpm]**					
	Systematic differences		Proportional differences		Linear model validity
Measure	Intercept A	95% CI	Slope B	95% CI	Cusum test for linearity
LOS	0.553	−15.895 to 16.538	0.979	0.881 to 1.075	*p* = 0.96
LSS	−30.747	−51.283 to −8.353	1.163	1.036 to 1.280	*p* = 0.46
DOS	−5.780	−34.600 to 30.700	1.020	0.814 to 1.187	*p* = 0.58
DSS	−3.000	−28.025 to 38.921	1.000	0.769 to 1.133	*p* = 0.39

A bootstrap confidence interval (1000 iterations; random number seed: 978). LOS, lateral open stance; LSS, lateral square stance; DOS, diagonal-in open stance; DSS, diagonal-in square stance. 95% CI, 95% confidence interval (CI); *p*, *p*-value.

**Table 4:** Kruskal–Wallis and Dunn post hoc tests for comparisons of each of those dependent variables (ball speed, accuracy and heart rate) between the two types of stances, tactical situation and flexibility level of the players. Df, degree of freedom; z, value of the z-statistic; W_i_, mean ranking of the first level/group of the comparison; W_j_, mean ranking of the second level/group of the comparison; *p*_holm_, Holm’s corrected *p*-value for multiple comparisons.

Ball Speed								
Kruskal-Wallis Test				Dunn Post Hoc Tests				
Factor	Statistic	df	*p*-Value	Comparison	z	W_i_	W_j_	*p*_holm_
Stance	6.858	1	0.009	Open–Square	−2.619	72.793	92.207	0.009
Situation	0.363	1	0.547	Diagonal–Lateral	−0.602	80.213	84.679	0.547
Gender	4.789	1	0.028	F–M	−2.190	72.853	89.333	0.028
Flexibility	3.991	1	0.046	Good–Low	1.998	90.276	75.448	0.046
**Accuracy Ratio**								
Kruskal-Wallis test				Dunn Post Hoc Tests				
Factor	Statistic	df	*p*-value	Comparison	z	W_i_	W_j_	*p*_holm_
Stance	0.314	1	0.575	Open–Square	−0.560	80.439	84.561	0.575
Situation	9.573	1	0.002	Diagonal–Lateral	3.094	94.162	71.393	0.002
Gender	6.817	1	0.009	F–M	2.611	93.912	74.417	0.009
Flexibility	11.339	1	<0.001	Good–Low	3.367	95.506	70.703	<0.001
**Max Heart Rate**								
Kruskal-Wallis test				Dunn Post Hoc Tests				
Factor	Statistic	df	*p*-value	Comparison	z	W_i_	W_j_	*p*_holm_
Stance	6.858	1	0.467	Open–Square	−0.728	73.901	79.099	0.467
Situation	0.363	1	0.121	Diagonal–Lateral	1.552	82.340	71.244	0.121
Gender	4.789	1	0.020	F–M	2.329	86.516	69.600	0.020
Flexibility	3.991	1	0.005	Good–Low	−2.786	65.736	85.689	0.005

**Table 5:** Medians of ball speed (km/h), accuracy ratios (number of valid target/totals), and maximum heart rate (bpm) achieved in performing the exercises in the four tactical situations: LOS; LSS; DOS, and DSS. The effect size is given by the matched rank biserial correlation.

**Ball speed (km/h)**							
Measure	*n.*	Median	IQR	Min–Max	Wilcoxon *p*-Value	Effect Size	SE
LOS	42	116	17	85–154	*p* < 0.001	−0.910	0.179
LSS	42	122	16	95–157			
DOS	40	114	19	76–152	*p* < 0.001	−0.922	0.182
DSS	40	118	19	79–157			
**Accuracy ratios (n. of valid targets/total)**							
Measure	*n.*	Median	IQR	Min–Max	Wilcoxon *p*-value	Effect Size	SE
LOS	42	0.30	0.19	0.05–0.55	*p* > 0.05	−0.218	0.189
LSS	42	0.35	0.10	0.10–0.55			
DOS	40	0.35	0.16	0.20–0.70	*p* > 0.05	0.103	0.197
DSS	40	0.40	0.15	0.10–0.75			
**Maximum heart rate (bpm)**							
Measure	*n.*	Median	IQR	Min–Max	Wilcoxon *p*-value	Effect Size	SE
LOS	39	172	15	125–193	*p* < 0.05	−0.482	0.189
LSS	39	174	18	124–194			
DOS	35	177	14	155–198	*p* > 0.05	−0.392	0.200
DSS	35	176	16	154–199			

LOS, and lateral open stance; LSS, lateral square stance; DOS, diagonal-in open stance; DSS, diagonal-in square stance. N., number of valid data; IQR, inter-quartile range; Min–Max, minimum and maximum; Wilcoxon *p*-value, Wilcoxon signed-rank *t*-test *p*-value; Effect Size, Effect Size for the Wilcoxon test; SE, Standard error of the effect size.

## Data Availability

The datasets supporting the conclusions of this article are included within the article. The raw data will be made available by the authors on request.

## Supplementary Materials

The following supporting information can be downloaded at: https://www.mdpi.com/article/10.3390/jfmk10020215/s1, Table S1: Maximum heart rate; Table S2: Ball speed and accuracy ratio.

## Abbreviations

The following abbreviations are used in this manuscript:

LSS	Lateral Square Stance
LOS	Lateral Open Stance
DSS	Diagonal-inside Square Stance
DOS	Diagonal-Inside Open Stance
