# Supplementary material for: Three Reasons for Playing the Tennis Forehand in Square Stance
Source: J Funct Morphol Kinesiol. 2025 Jun 5;10(2):215. doi: 10.3390/jfmk10020215 (PMC12194768; doi:10.3390/jfmk10020215)
Supplement: Supplementary file 1 [file jfmk-10-00215-s001.zip › jfmk-3575127-supplementary.pdf]

Supplementary material

Table S1. Maximum heart rate (bpm) achieved in performing the exercises in the four tactical situations (data expressed as the median value of executions).

	Lateral		Diagonal-In			Lateral		Diagonal-In	
**ID**	**Open**	**Square**	**Open**	**Square**	**ID**	**Open**	**Square**	**Open**	**Square**
#1	167.0	170.5	175.0	153.5	#22	177.5	181.5	--	--
#2	176.0	183.0	184.5	189.5	#23	161.5	163.0	--	--
#3	173.5	176.5	176.5	172.0	#24	181.0	179.0	178.0	174.0
#4	171.5	174.0	159.0	168.5	#25	185.0	185.5	188.0	188.0
#5	176.5	177.0	180.0	175.5	#26	163.0	164.0	172.5	175.0
#6	--	--	--	--	#27	173.0	175.0	175.0	176.5
#7	--	--	165.5	165.0	#28	--	--	--	--
#8	133.0	123.5	--	--	#29	192.5	194.0	194.5	199.0
#9	160.0	187.5	185.0	186.5	#30	184.5	186.5	189.0	189.0
#10	164.0	160.0	181.0	183.5	#31	177.5	179.5	179.0	184.0
#11	173.0	176.5	170.5	176.5	#32	181.5	185.5	188.0	188.0
#12	178.0	172.5	167.0	164.5	#33	168.0	168.0	172.0	173.0
#13	173.5	178.0	179.0	181.5	#34	182.5	183.5	179.5	182.5
#14	179.0	180.5	177.0	181.5	#35	155.5	160.0	166.5	168.0
#15	151.0	154.5	154.5	160.5	#36	184.0	188.5	179.5	182.0
#16	168.0	168.0	170.5	166.0	#37	167.0	166.5	167.0	171.0
#17	166.5	169.0	161.0	164.0	#38	169.0	167.0	164.0	168.0
#18	164.5	164.5	162.0	163.5	#39	186.0	188.5	198.0	198.5
#19	148.5	143.5	--	--	#40	171.5	172.0	180.0	179.0
#20	159.5	158.0	161.0	162.5	#41	125.0	131.0	--	--
#21	158.0	159.5	163.0	160.5	#42	191.0	190.0	182.5	181.5

Table S2. Ball speed (km/h) and accuracy ratio of the shoot in the four different tactical situations: LOS, and lateral open stance; LSS, lateral square stance; DOS, diagonal-in open stance; DSS, diagonal-in square stance.

	Lateral				Diagonal-In			
	Speed [km/h] Median (IQR)		Accuracy ratio		Speed [km/h] Median (IQR)		Accuracy ratio	
**Player ID**	**Open**	**Square**	**Open**	**Square**	**Open**	**Square**	**Open**	**Square**
#1	85.5 (14)	95.0 (5)	0.30	0.20	76.0 (6)	79.0 (10)	0.35	0.40
#2	88.5 (14)	104.0 (10)	0.05	0.55	103.0 (12)	107.0 (11)	0.25	0.30
#3	108.0 (9)	113.0 (9)	0.35	0.45	100.5 (13)	109.0 (6)	0.45	0.25
#4	110.0 (9)	121.0 (5)	0.50	0.30	108.0 (8)	116.0 (12)	0.35	0.35
#5	112.5 (11)	129.0 (8)	0.45	0.35	125.5 (12)	132.0 (8)	0.70	0.40
#6	131.0 (9)	135.0 (12)	0.25	0.50	114.0 (14)	127.0 (6)	0.55	0.50
#7	122.0 (12)	128.0 (11)	0.45	0.50	109.0 (13)	116.0 (4)	0.25	0.40
#8	121.0 (9)	124.0 (7)	0.25	0.35	104.0 (11)	111.0 (17)	0.45	0.60
#9	105.5 (10)	110.0 (6)	0.20	0.30	105.0 (6)	109.0 (7)	0.55	0.30
#10	103.0 (5)	111.0 (3)	0.15	0.10	105.0 (4)	116.0 (7)	0.20	0.30
#11	113.0 (7)	126.5 (8)	0.30	0.50	115.0 (5)	122.0 (6)	0.30	0.30
#12	116.0 (8)	124.0 (11)	0.50	0.40	116.0 (4)	122.0 (7)	0.30	0.50
#13	108.0 (6)	109.0 (5)	0.25	0.30	104.0 (11)	113.0 (6)	0.35	0.20
#14	119.0 (9)	127.0 (7)	0.30	0.20	116.0 (8)	124.0 (11)	0.35	0.45
#15	98.0 (9)	110.0 (7)	0.20	0.20	91.5 (13)	97.0 (9)	0.33	0.45
#16	131.0 (14)	131.0 (10)	0.35	0.35	128.0 (10)	127.0 (12)	0.45	0.35
#17	114.0 (6)	121.0 (13)	0.40	0.35	112.0 (7)	118.0 (6)	0.60	0.65
#18	116.5 (7)	117.0 (6)	0.30	0.25	111.0 (5)	117.0 (9)	0.30	0.40
#19	154.0 (9)	157.0 (12)	0.30	0.25	152.0 (7)	157.0 (12)	0.40	0.20
#20	140.0 (8)	145.0 (10)	0.30	0.45	132.0 (8)	142.0 (9)	0.35	0.45
#21	134.0 (5)	139.0 (13)	0.20	0.35	139.0 (14)	137.0 (8)	0.30	0.15
#22	119.0 (5)	116.0 (7)	0.40	0.35	--	--	--	--
#23	128.0 (7)	131.0 (6)	0.50	0.55	129.0 (3)	137.0 (5)	0.60	0.50
#24	121.0 (6)	122.0 (7)	0.25	0.25	121.0 (2)	120.5 (12)	0.40	0.40
#25	120.5 (9)	124.0 (9)	0.35	0.30	133.0 (12)	135.0 (4)	0.30	0.30
#26	116.0 (10)	123.0 (9)	0.30	0.45	128.5 (11)	134.0 (6)	0.40	0.55
#27	133.5 (7)	138.0 (9)	0.50	0.35	136.0 (8)	144.0 (9)	0.35	0.30
#28	108.0 (6)	116.0 (6)	0.35	0.35	107.0 (11)	117.0 (11)	0.50	0.45
#29	114.0 (6)	117.0 (5)	0.35	0.40	113.5 (9)	113.5 (10)	0.35	0.45
#30	109.5 (15)	119.0 (7)	0.20	0.25	125.0 (5)	133.0 (6)	0.25	0.15
#31	113.5 (9)	113.5 (11)	0.55	0.30	111.0 (4)	116.5 (7)	0.40	0.40
#32	104.0 (7)	111.0 (7)	0.20	0.40	109.0 (6)	108.5 (9)	0.55	0.45
#33	119.0 (14)	126.5 (14)	0.30	0.10	113.5 (11)	114.0 (6)	0.50	0.25
#34	119.0 (6)	121.0 (4)	0.40	0.40	105.5 (5)	110.0 (5)	0.35	0.45
#35	113.5 (7)	121.0 (6)	0.45	0.20	108.5 (7)	120.0 (9)	0.60	0.50
#36	138.0 (10)	137.5 (13)	0.45	0.40	130.0 (6)	133.5 (15)	0.50	0.45
#37	112.0 (4)	111.0 (7)	0.10	0.35	109.0 (8)	107.5 (6)	0.20	0.40
#38	126.0 (6)	123.0 (10)	0.20	0.40	116.0 (3)	117.0 (3)	0.40	0.40
#39	136.0 (9)	139.0 (5)	0.30	0.30	135.0 (7)	142.0 (7)	0.45	0.45
#40	133.0 (10)	130.0 (4)	0.25	0.35	124.0 (10)	127.0 (5)	0.30	0.10
#41	97.0 (7)	101.0 (6)	0.20	0.30	--	--	--	--
#42	100.0 (12)	109.0 (15)	0.15	0.35	115.5 (13)	113.5 (19)	0.35	0.25
